# Effectiveness of magnetocardiography as a non-invasive tool for functional assessment of myocardial ischemia in patients with stable coronary artery disease

**DOI:** 10.3389/fmedt.2025.1611046

**Published:** 2025-07-22

**Authors:** Wen-fei He, Li-huan Zeng, Nan-shan Xie, Hao-xing Liu, Wen-min Cui, Ying Wang, Zhi-jian Zhang, Guan-lun Ye, Zhi-ying Qin, Zhi-qiang Guo, Jun Ma, Jian-fang Luo

**Affiliations:** ^1^ Department of Cardiology, Guangdong Provincial People’s Hospital’s Nanhai Hospital, the Second People’s Hospital of Nanhai District, Foshan, China; ^2^ Department of Cardiology, Guangdong Cardiovascular Institute, Guangdong Provincial People’s Hospital, Guangdong Academy of Medical Sciences, Guangzhou, China; ^3^ Guangdong Provincial Key Laboratory of Coronary Heart Disease Prevention, Guangdong Cardiovascular Institute, Guangdong Provincial People’s Hospital (Guangdong Academy of Medical Sciences), Southern Medical University, Guangzhou, China; ^4^ Department of General Practice, Guicheng Community Health Service Center of Nanhai District, Foshan, China; ^5^ R&D Department, Shenzhen Raysight Intelligent Medical Technology Corporation, Shenzhen, Guangzhou, China

**Keywords:** magnetocardiography (MCG), angiography-derived fractional flow reserve, stable coronary artery disease (SCAD), coronary ischemia, assessment

## Abstract

**Background:**

Identification of coronary ischemia in suspected coronary artery disease (CAD) remains challenging. Magnetocardiography (MCG) demonstrated comparably high diagnostic efficiency for detecting coronary ischemia in previous studies. However, limited evidence exists comparing MCG vs. computed tomography angiography-derived fractional flow reserve (CTFFR) in suspected CAD patients.

**Methods:**

A total of 291 patients with CTA-confirmed diameter stenosis ranging from 30% to 90% were included and divided into two groups based on the CTFFR values, the stable coronary artery disease (SCAD) group (≤0.8) and the non-SCAD group (>0.8). Magnetic field map (MFM) parameters were employed to construct a diagnostic model. The performance of the models was evaluated using receiver operating characteristic (ROC) curves, accuracy, sensitivity, specificity, positive predictive value (PPV), and negative predictive value (NPV).

**Results:**

Patients with SCAD showed a mean MCG score of 5.6 ± 2.9, while the non-SCAD group demonstrated a mean score of 2.0 ± 1.9 (*P* < 0.001). The area under the curve (AUC) for ROC analysis was 0.824 (95% CI: 0.772–0.877). Point 5 was selected as the operational cutoff value providing a favorable balance of sensitivity and specificity. Ultimately, MCG score yielded a sensitivity of 69.6%, specificity of 87.9%, PPV of 72.7%, NPV of 86.2%, and accuracy of 82.1%.

**Conclusions:**

Compared to CTFFR, MCG demonstrated superior specificity and moderate sensitivity for detecting CAD in patients with diameter stenosis CTA ranging from 30% to 90%. It may provide an alternative to functional evaluation prior to invasive or radiation exposure methods.

## Highlights

• What is already known on this topic – Currently, certain diagnostic measures with inherent limitations are employed to diagnose SCAD. MCG is established as an efficient tool for cardiac disease diagnosis. An evidence gap persists regarding MCG vs. CTFFR in assessing myocardial ischemia.
• What this study adds – Compared with CTFFR, MCG demonstrated superior specificity and moderate sensitivity for detecting CAD in patients with CTA-confirmed diameter stenosis ranging from 30% to 90%.
• How this study might affect research, practice, or policy - MCG is a simple and effective method for detecting myocardial ischemia.

## Introduction

Coronary artery disease (CAD) remains the leading cause of death worldwide and is associated with substantial individual, economic, and societal burdens. Only a minority of patients present mild symptoms in stable coronary artery disease (SCAD) (1). To lower the coronary deaths in asymptomatic adults, numerous measurements of risk factors and markers, as well as stress tests, are employed as screening investigations.

Fractional flow reserve (FFR) assesses physiological stenosis severity. However, coronary angiography in low-risk patients imposes unnecessary burden and risk, while, FFR, which requires specialized pressure guidewires, is both technically complex and costly. Routine electrocardiogram and echocardiography demonstrate relatively high diagnostic performance for acute myocardial infarction, but are insufficient for SCAD. Although the exercise treadmill testing, stress echocardiography, cardiac magnetic resonance imaging (CMR) and single-photon emission computed tomography (SPECT) can also evaluate myocardial ischemia, they cannot assess coronary artery stenosis, moreover, these methods present several limitations, including complex procedures, high technical demands, substantial costs, and potential radiation exposure (2, 3). Therefore, a non-invasive, safe and simple method is required to facilitate the early diagnosis of stable coronary artery disease.

Computed tomography angiography-derived fractional flow reserve (CTFFR) is a novel noninvasive approach for precisely localizing ischemia-causing coronary stenosis. It utilizes computational fluid dynamics (CFD) to calculate “3-vessel” FFR from standard coronary computed tomography angiography (CCTA) images without requiring additional imaging or vasodilators (4, 5). Furthermore, the PLATFORM study demonstrates that CTFFR enhances diagnostic certainty by reducing invasive coronary angiography cancellations by 61% and significantly lowering the rate of detecting non-obstructive CAD (6).

Magnetocardiography (MCG) measures changes in the heart's electromagnetic field, which are altered in the diseased hearts, likely due to modifications in the trajectory or number of moving ions or electrons. An objective scoring system facilitates interpretation and comparison of results. Given the absence of radiation, MCG is ideal for screening and frequent monitoring of heart conditions, especially in CAD (7, 8). MCG detects minute electrophysiological changes that lead to abnormal currents, especially occurring in early ischemia, enabling early CAD diagnosis (9).

Nowadays, MCG has shown certain potential in the diagnosis of CAD (10), arrhythmia and inflammatory cardiomyopathy (11), and has become a research hotspot worldwide. However, most of the relevant studies are exploratory studies, with a small sample size and lacking in-depth research. Also, the performance of MCG in patients with relatively low risk of myocardial ischemia during routine check-ups is still inconclusive. This study aims to use the latest MCG equipment to assess myocardial ischemic function in patients with stable coronary artery disease.

## Methods

### Study design and population

This was a single-center, prospective, observational cohort study that consecutively enrolled 355 patients with suspected SCAD at Guangdong Provincial People's Hospital's Nanhai Hospital, comprising 281 male patients and having a mean age of 52.3 years. To evaluate the accuracy of MCG in detecting hemodynamically significant SCAD during routine check-ups, CTFFR was utilized as the reference standard for non-invasive examinations.

**Figure 1:**
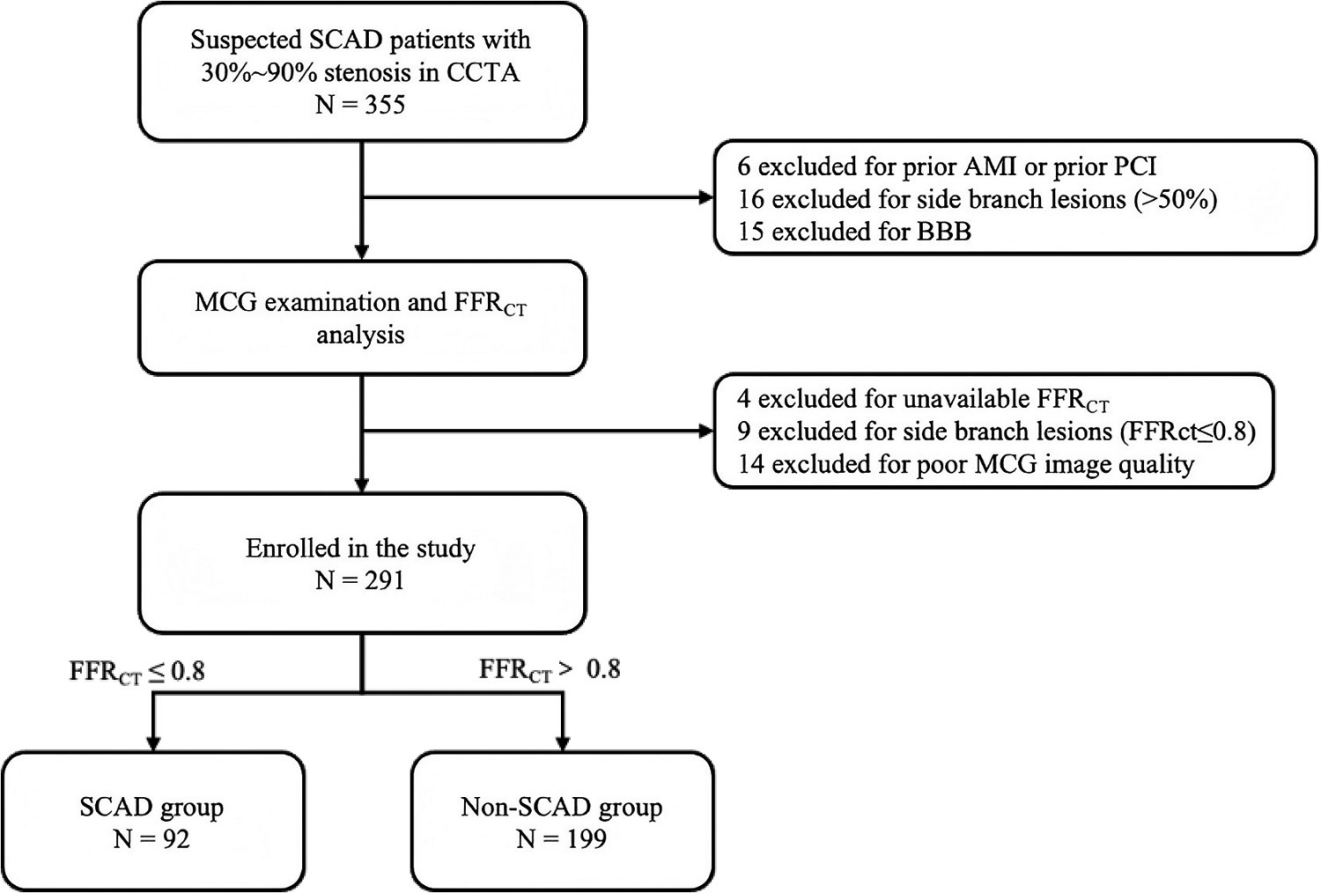
Flow chart of the study. SCAD, stable coronary artery disease; CCTA, coronary computed tomography angiography; MCG, magnetocardiography; CTFFR, CTA-derived fractional flow reserve; AMI, acute myocardial infarction; PCI, percutaneous coronary intervention; BBB, bundle branch block.

For adults with cardiovascular risk factors or suspected symptoms during routine check-ups, CCTA was used as the primary screening tool for early-stage SCAD. Patients with 30% to 90% diameter stenosis confirmed by CCTA in at least one major epicardial coronary artery were prospectively enrolled in this study. Generally, patients with >90% stenosis on CCTA have a clear clinical indication for invasive coronary angiography, while those with <30% stenosis can be safely ruled out from severe myocardial ischemia (12). To ensure the accuracy, safety, and specificity of detection, patients who had a history of any of the following conditions, including revascularization, acute myocardial infarction, hypertrophic or dilated cardiomyopathy, or complete bundle branch block, were excluded from the study. Additionally, notably, people with implanted metallic devices (e.g., pacemaker, ICDs) or inability to lie flat—precluding MCG examination—were excluded from recruitment. Patients with main coronary artery lesions and side branch lesions were excluded. After enrollment, all the patients underwent the MCG examination and CTFFR analysis before subsequent medical or interventional therapy. Those with CTFFR ≤0.8 were categorized into the SCAD group, while the remaining subjects comprised the control group. Other related clinical data were extracted from the hospital information system. The flow chart outlining the study process is presented in Figure 1.

### MCG examination

**Figure 2:**
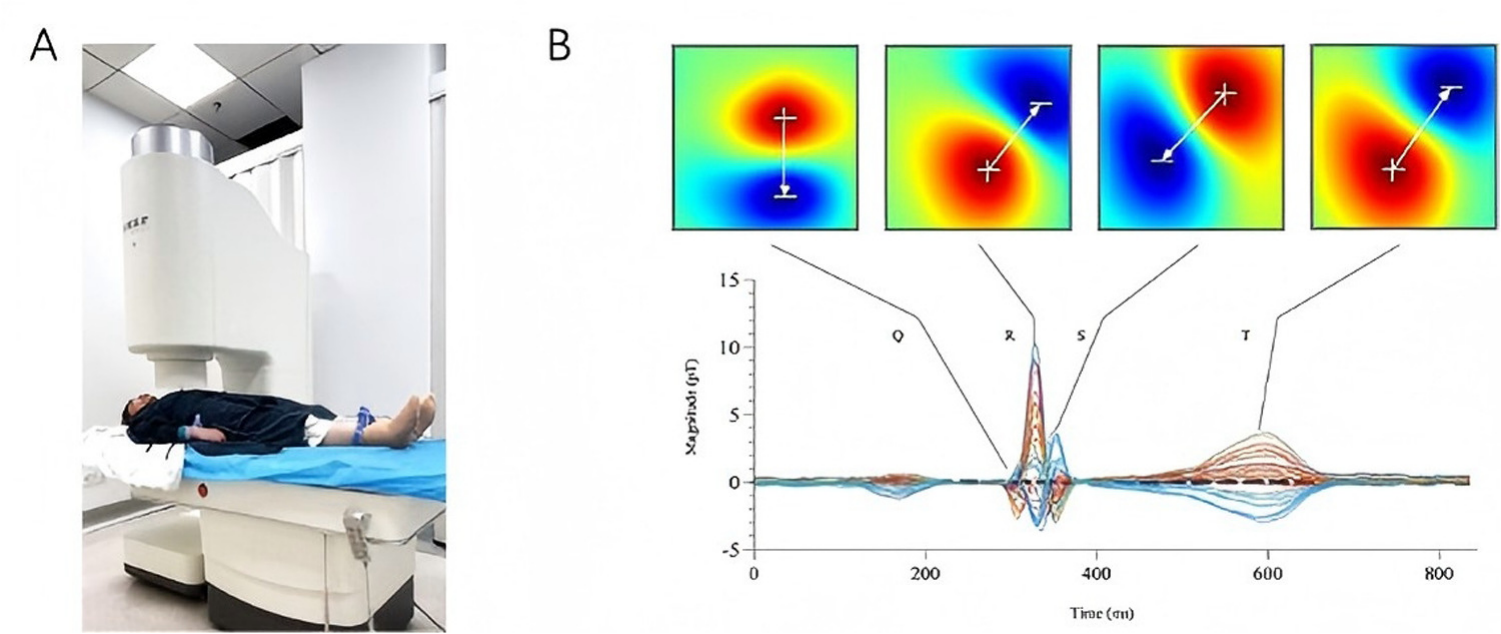
MCG equipment and magnetic field maps. **(A)**: the MCG system used in this study (MD-U0-92001, Shanghai MEDI Instruments Ltd., Shanghai, China); **(B)**: magnetic field maps of the Q, R, S, and T waves based on averaged signals.

The MCG scans were performed by an unshielded 9-channel MCG system (MD-U0-92001, Shanghai MEDI Instruments Ltd., Shanghai, China) as illustrated in Figure 2A. This equipment utilized superconducting quantum interference device (SQUID) sensors linked to second-order axial gradiometers to detect the extremely weak magnetic field generated by electrical activity of the heart. After removing all jewelry, electronic devices, and other metal objects, we sequentially acquire magnetic field signals at four distinct positions over the precordial region: superior right, superior left, inferior left, inferior right. The composite data from these four acquisition positions collectively generates a 36-point measurement grid. The whole examination process lasted for approximately 5 min at a sampling rate of 1,000 Hz, with simultaneous recording of the ECG lead-II as reference. The raw signals were then baseline-corrected, digitally filtered, and averaged to increase the signal-to-noise ratio. Magnetic field maps (MFMs) were digitally plotted based on the iso-field contour maps at each millisecond of one cardiac cycle (Figure 2B). 8 parameters derived from 5 categories of MFM features were evaluated for each patient. The validity of these 5 feature categories has been established in prior research, with Cui et al. demonstrating their efficacy in predicting the risk of severe coronary artery stenosis among patients presenting with angina-like symptoms (13). In this study, however, we aimed to investigate whether these parameters could be applied to general screening populations.

### CCTA assessment

The CCTA images [Revolution HD (GE)] were parsed by two radiologists with more than five years of experience, and the location of the target lesion on CCTA was demarcated jointly by the radiologists and the cardiologist. The percentage diameter stenosis of the target lesion was quantified using offline quantitative coronary CT angiography software (Syngo·via, Siemens Healthineers, Forchheim, Germany). Curved multiplanar reconstructions, maximum intensity projections, and volume rendering techniques were employed to generate diagnostic images for interpretation (14).

### CTFFR measurement

CTFFR analysis was performed using a dedicated software (RuiXin-CTFFR, Raysight Inc., Shenzhen, China). This software employs CFD to calculate CTFFR values based on the CCTA images. First, the initial segmentation model of the entire coronary artery was extracted, from which each coronary artery centerline and contour were obtained by the region growth algorithm. The extracted coronary artery contours were subsequently connected and smoothed to facilitate the reconstruction of a three-dimensional (3D) model. Using the morphological data extracted from the CT imaging and a statistical prediction model incorporating the patient's baseline characteristics (including allometric growth principles), patient-specific physiological parameters were determined. An unstructured mesh was then generated on the 3D coronary model, with the blood flow modeled as an incompressible Newtonian fluid. The incompressible Navier-Stokes equations were subsequently solved using a finite element algorithm, enabling the calculation of pressure and velocity values at each grid point within the 3D coronary model, ultimately yielding the final CTFFR measurements (15).

### Statistical analysis

With *α* = 0.025 and *β* = 0.1, based on previous literature reporting a diagnostic accuracy of 85% for the CTFFR examination (16), we set the non-inferiority margin at 5%, meaning that the diagnostic accuracy of MCG should be at least 80%. It was calculated that at least 169 samples are required to undergo both MCG and CT-FFR examinations. Considering the dropout rate of 20%, it was finally estimated that at least 211 samples are required for this clinical trial.

Continuous variables were expressed as means ± standard deviations (SD). For normally distributed measurement data, *T*-test was used to compare the differences between the two groups. For non-normally distributed measurement data, Mann–Whitney *U*-test was used for group comparisons. Count data were expressed as the number of cases and composition ratio (%), and the chi-square test was used for comparison between groups. The diagnostic value of MCG score was assessed using receiver operating characteristic (ROC) curve analysis. The optimal cutoff value for the score was determined by Youden's J Index, defined as: Youden's J Index = Sensitivity + Specificity - 1. Statistical analysis evaluation was conducted using SPSS 23.0 and R 4.2.2 software. A significance level of *P* < 0.05 was considered statistically significant.

### Ethics declarations

The study was approved by the Hospital Ethics Committee and registered at https://www.ClinicalTrails.gov (NCT06123728). Written informed consents were obtained from each patient before enrollment.

## Results

### Patient characteristics

Among 355 patients with 30%–90% diameter stenosis on CCTA between April 2023 and December 2023, 21 patients were excluded based on medical history (6 had prior AMI or prior PCI, 15 had a bundle branch block). Additionally, 4 patients were unavailable for CTFFR analysis and 14 patients had uninterpretable MCG data due to noise interference, these 18 patients were also excluded. Furthermore, 25 patients were excluded due to side branch lesions. Ultimately, a total of 291 patients were recruited. All the patients were categorized into two groups based on their CTFFR (threshold ≤0.80): the SCAD group (*N* = 92) and the non-SCAD group (*N* = 199) (Figure 1).

The baseline characteristics are summarized in Table 1. The median age (53 years vs. 51 years, *P* = 0.012) and the median BMI (26.51 kg/m^2^ vs. 25. 10 kg/m^2^, *P* = 0.022) were higher in the SCAD group compared to the non-SCAD group.

**Table 1:** Baseline characteristics of the study population.

Characteristic	Overall (*N* = 291)	SCAD (*N* = 199)	Non-SCAD (*N* = 92)	*P*-value
Male, *n* (%)	281 (96.6)	192 (96.5)	89 (96.7)	1
Age (years)	52.3 ± 4.8	51.8 ± 4.3	53.4 ± 5.6	0.012
BMI (kg/m^2^)	25.8 ± 3.6	25.5 ± 3.7	26.4 ± 3.3	0.022
Typical symptoms, *n* (%)	34 (13.1)	24 (13.1)	10 (13.0)	0.987
Current smoker, *n* (%)	150 (54.9)	102 (54.3)	48 (56.5)	0.834
Hypertension, *n* (%)	104 (38.1)	57 (30.3)	47 (55.3)	<0.001
Hyperlipidemia, *n* (%)	100 (36.6)	73 (38.8)	27 (31.8)	0.324
Diabetes, *n* (%)	44 (16.1)	27 (14.4)	17 (20.0)	0.32
MCG score	3.2 ± 2.8	5.6 ± 2.9	2.0 ± 1.9	<0.001

SCAD, stable coronary artery disease.

Hypertension was more prevalent in the SCAD group (55.3% vs. 30.3%, *P* < 0.001). Other clinical characteristics showed no significant differences between the two groups.

### Performance of MCG score

**Figure 3:**
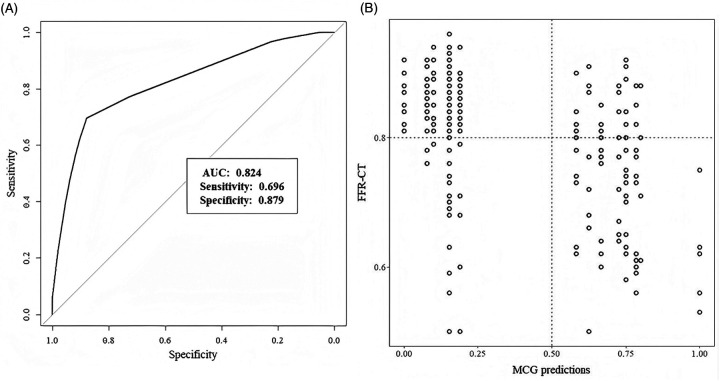
**(A)** Performance of MCG score. Receiver-operating characteristic curves. **(B)** Scatter plot ofCTFFR values and MCG predictions.

Patients with SCAD showed a mean MCG score of 5.6 ± 2.9, while the non-SCAD group had a mean score of 2.0 ± 1.9 (*P* < 0.001). The area under the curve (AUC) for ROC analysis was 0.824 (95% CI: 0.772–0.877), shown in Figure 3. By comparing the Youden's J Index across all score points, we identified 1.89 as the cutoff value with the maximum Youden's J Index. However, given the considerable gap between this optimal cutoff and the next higher available threshold (5.83), we selected point 5 as the operational cutoff, which provided favorable sensitivity-specificity balance. MCG score achieved a sensitivity of 69.6%, specificity of 87.9%, PPV of 72.7%, NPV of 86.2%, and accuracy of 82. 1%. The performance metrics of MCG score are summarized in Table 2.

**Table 2:** The performance of the MCG score.

Parameter	Estimated value	95% confidence Interval
AUC	0.824	(0.772–0.877)
SEN	0.696	(0.590–0.785)
SPE	0.879	(0.824–0.920)
PPV	0.727	(0.620–0.814)
NPV	0.862	(0.805–0.905)
ACC	0.821	(0.772–0.864)

AUC, area under the curve; SEN, sensitivity; SPE, specificity; NPV, negative predictive value; PPV, positive predictive value; ACC, accuracy.

### MCG results in different subgroups

The degree of stenosis in each major epicardial coronary artery on CCTA and the CTFFR values per vessel are presented in Table 3. The prevalence of obstructive CAD by CCTA differed substantially from the prevalence of ischemia by CTFFR. 132(45.4%) patients had at least one vessel with ≥50% diameter stenosis, while only 92 (31.6%) patients exhibited CTFFR ≤0.8. Furthermore, LAD was the most prevalent lesion site, involving 95 patients with stenosis ≥50% and 77 patients with CTFFR ≤0.80.

**Table 3:** Vessel characteristics by CCTA and FFR_CT_.

Vessel characteristic	LAD, *n* (%)	LCX, *n* (%)	RCA, *n* (%)	The most severe vessel, *n* (%)
CCTA results				
Stenosis <50	196 (67.4)	254 (87.3)	247 (84.9)	159 (54.6)
50 ≤stenosis <70	69 (23.7)	30 (10.3)	32 (11.0)	97 (33.3)
Stenosis ≥70	26 (8.9)	7 (2.4)	12 (4.1)	35 (12.0)
FFR_CT_ results				
FFR_CT_ >0.8	214 (73.5)	271 (93.1)	265 (91.1)	199 (68.4)
FFR_CT_ ≤0.8	77 (26.5)	20 (6.9)	26 (8.9)	92 (31.6)

CCTA, coronary computed tomography angiography; FFR_CT_, computed tomography angiography-derived fractional flow reserve; LAD, left coronary artery; LCX, left circumflex artery; RCA, right coronary artery.

Compared with CCTA results, the concordance rates of CTFFR and MCG with coronary stenosis were 78.7% (229/291) and 66.3% (193/291), respectively. In contrast, CTFFR results showed greater concordance with MCG evaluation, with a high concordance rate of 82.1% (239/291). Among patients with CCTA stenosis <50%, 133 individuals (83.6%) had concordant CTFFR and MCG results. Conversely, among patients with CCTA stenosis ≥50%, 106 individuals (80.3%) had concordant CTFFR and MCG results (Table 4).

**Table 4:** MCG results in different subgroups of CCTA and FFR_CT_.

Subgroup	MCG (−), *n*	MCG (+), *n*
Stenosis <50% & FFR_CT_ >0.8	127	21
Stenosis < 50% & FFR_CT_ ≤0.8	5	6
Stenosis ≥50% & FFR_CT_ >0.8	48	3
Stenosis ≥50% & FFR_CT_ ≤0.8	23	58

CCTA, coronary computed tomography angiography; FFR_CT_, computed tomography angiography-derived fractional flow reserve.

## Discussion

To the best of our knowledge, this was the first study to evaluate the diagnostic performance of the MCG against CTFFR. The main finding of our analysis was that, compared to CTFFR, MCG showed high sensitive and specific for predicting myocardial ischemia in patients with cardiovascular risk factors or suspected symptoms who had 30%–90% diameter stenosis on CCTA.

MCG is a non-contact, non-invasive, non-radiation method to detect myocardial ischemia by recording magnetic fields generated by cardiac electrical activity (17). Previous studies confirmed that MCG demonstrates high sensitivity and specificity, compared to other diagnostic modalities, such as electrocardiography (ECG), SPECT, and FFR (18–20). An evidence gap exists regarding the diagnostic value of CAD between MCG and CTFFR. Additionally, nearly all the studies enrolled high-risk patients with CTA- or angiographically-confirmed CAD, or classic with angina pectoris, while evidence for MCG in low-risk patients remains limited (21, 22). Lastly, when interpreting previous results, small sample size limitations cannot be overlooked (23). Thus, conducting this study to evaluate MCG's diagnostic performance against CTFFR in patients with 30%–90% diameter stenosis on CCTA was meaningful.

In the current study, we achieved a sensitivity of 69.6%, specificity of 87.9%, PPV of 72.7%, NPV of 86.2%, and accuracy of 82. 1%. Our finding was different from many previous studies considering MCG as a highly sensitive but less specific method (24–26). First, low-risk suspected CAD patients comprised most enrolled participants. Patients in our study had low rates of typical symptoms, hypertension, hyperlipidemia, and diabetes. However, when reviewing previous studies, the high risks mentioned above were considerably prevalent (24–26). Second, the coronary artery stenosis was less severe than other studies (26, 27). There were 132 patients with coronary stenosis ≥50% but only 92 patients with CTFFR ≤0.8. Concurrently, the scatter plot of CTFFR values and MCG predictions showed us most CTFFR values >0.60. Thus, our cohort represented an unselected population, better reflecting real-world research. Based on these results, we believe that MCG could be a promising screening tool for individuals with low risk of CAD.

In addition to our study, other studies have also confirmed that MCG exhibits higher specificity and lower sensitivity. Van Leeuwen et al. found that MCG had a sensitivity of 85% in CAD patients with prior myocardial infarction vs. 68% in those without, and a specificity of 90% (21). Fenici et al. reported that two machine-learning techniques combined to identify abnormal MCG ventricular repolarization (VR) in 25 IHD patients and exclude VR abnormalities in 28 controls, yielding 75% sensitivity and 85% specificity (28). The results of these studies are consistent with our findings. In clinical practice, high specificity can guide decisions for further angiography, reducing the frequency of invasive procedures.

According to the subgroup result of CTFFR and MCG, for patients whose CTFFR was >0.8 and MCG was positive, the discrepancy may be explained by the incomplete correspondence between hemodynamic alterations in major coronary branches and the state of myocardial injury caused by ischemia. In SCAD, hemodynamic alterations may exhibit a temporal delay in inducing myocardial damage and electrophysiological alterations. During this subclinical phase, CTFFR may detect hemodynamic abnormalities while MCG remains negative due to the absence of established tissue injury. Furthermore, hemodynamic abnormalities in major coronary vessels are not the sole cause of myocardial ischemia. Coronary microvascular dysfunction (CMVD)—a structural and functional disorder affecting small arteries, arterioles, and capillaries—frequently induces myocardial ischemia despite normal hemodynamics in major coronary branches (29, 30), in the study by Quesada et al., MCG demonstrated the capability to detect CMVD through a 90-second non-invasive scan, eliminating the need for intravenous vasodilators or ionizing radiation (31). In such scenarios, CTFFR may yield negative results, whereas MCG could demonstrate positive findings.

In the present study, an approximately 21.3% rate of mismatch between CCTA and CTFFR was found. Among these mismatched data, MCG showed a positivity rate of 54.5% (6/11) with positive mismatch (CTFFR ≤0.8, CCTA <50%), while demonstrating a positivity rate of 5.9% (3/51) with negative mismatch (FFR>0.8, CCTA ≥50%), which indicated a high correlation between MCG and hemodynamically significant lesions. This is like the previous work by Park that MCG could accurately detect CAD vs. invasively determined FFR with a sensitivity of 86.7% and specificity of 73.9% (20). Considering the increasing need for early detection of ischemia in people at high risk of cardiovascular disease, MCG has been proposed as a noninvasive and contactless technique for functional diagnosis of the heart (32, 33). ECG relies on horizontal body surface potentials, making it vulnerable to signal cancellation that masks tangential/vortex currents. MCG directly measures perpendicular magnetic fields from cardiac activity. Tangential currents' magnetic fields are easily captured by sensors. Though electrically undetectable due to cancellation, vortex currents still generate measurable percardial magnetic fields via the right-hand rule (34, 35), MCG has the potential to benefit the assessment of suspected patients without CAD. Based on the application of artificial intelligence in the evaluation of disease, techniques like machine learning methods were implemented to automate diagnosis (36). In the previous study, back-propagation neural network (BNN) and direct kernel self-organizing map (DK-SOM) were applied to explore the IHD pattern recorded by MCG and exhibited accuracy of 74.5% and 80.4% respectively (37).

In terms of convenience and resource utilization, the implementation of an MCG pathway could significantly reduce patients' length of stay, and it just need 10 min to finish the assessment of the myocardial ischemia. Besides, compared to other measures, MCG yielded significantly higher patients' satisfaction (38, 39). Furthermore, given the substantial number of patients presenting with chest pain in outpatient and emergency departments, magnetocardiography (MCG) holds significant potential as a non-invasive diagnostic tool. When used complementarily to established modalities such as CCTA and SPECT (40, 41), MCG may effectively reduce both misdiagnosis rates and unnecessary exposure to surgical or other invasive procedures (42).

## Limitation

Several limitations should be acknowledged. Firstly, it was a single-center study conducted in China, the results might not be applied to whole population. Secondly, it is noted that the vast majority of patients in the current study were male. Though such a sex bias could not be ignored, the principle of MCG was to record magnetic fields generated by the electrical activity and there was no evidence revealing a difference in sex subgroup in the field of MCG. Thus, we recognized that sex would not change the final results. Thirdly, we were lack of the results of coronary angiography and FFR regarded as the golden standard of functional evaluation. Lastly, there are only 35 patients whose stenosis ≥70 confirmed by CCTA, the small sample of this subgroup might contribute to the low sensitivity.

## Conclusion

Compared to CTFFR, MCG yielded a good specificity and acceptable sensitivity for the detection of CAD in patients whose diameter stenosis CTA was confirmed from 30% to 90%. It may provide an alternative to functional evaluation before other invasive or radiation exposure methods. Further studies are warranted to determine the role of MCG in this context.

## Data Availability

The raw data supporting the conclusions of this article will be made available by the authors, without undue reservation.
